# Broccoli or Sulforaphane: Is It the Source or Dose That Matters?

**DOI:** 10.3390/molecules24193593

**Published:** 2019-10-06

**Authors:** Yoko Yagishita, Jed W. Fahey, Albena T. Dinkova-Kostova, Thomas W. Kensler

**Affiliations:** 1 Translational Research Program, Fred Hutchinson Cancer Research Center, Seattle, WA 98109, USA; yyagishi@fredhutch.org; 2 Department of Medicine, Division of Clinical Pharmacology, Johns Hopkins School of Medicine, Baltimore, MD 21205, USA; jfahey@jhmi.edu; 3 Department of Pharmacology and Molecular Sciences, Johns Hopkins School of Medicine, Baltimore, MD 21205, USA; 4 Cullman Chemoprotection Center, Johns Hopkins School of Medicine, Baltimore, MD 21205, USA; 5 Jacqui Wood Cancer Centre, Division of Cellular Medicine, Ninewells Hospital and Medical School, University of Dundee, Dundee DD1 9SY, Scotland DD1 9SY, UK; A.DinkovaKostova@dundee.ac.uk

**Keywords:** broccoli, sulforaphane, glucoraphanin, myrosinase, chemoprotection, allometric scaling, clinical trials, Nrf2, toxicity

## Abstract

There is robust epidemiological evidence for the beneficial effects of broccoli consumption on health, many of them clearly mediated by the isothiocyanate sulforaphane. Present in the plant as its precursor, glucoraphanin, sulforaphane is formed through the actions of myrosinase, a β-thioglucosidase present in either the plant tissue or the mammalian microbiome. Since first isolated from broccoli and demonstrated to have cancer chemoprotective properties in rats in the early 1990s, over 3000 publications have described its efficacy in rodent disease models, underlying mechanisms of action or, to date, over 50 clinical trials examining pharmacokinetics, pharmacodynamics and disease mitigation. This review evaluates the current state of knowledge regarding the relationships between formulation (e.g., plants, sprouts, beverages, supplements), bioavailability and efficacy, and the doses of glucoraphanin and/or sulforaphane that have been used in pre-clinical and clinical studies. We pay special attention to the challenges for better integration of animal model and clinical studies, particularly with regard to selection of dose and route of administration. More effort is required to elucidate underlying mechanisms of action and to develop and validate biomarkers of pharmacodynamic action in humans. A sobering lesson is that changes in approach will be required to implement a public health paradigm for dispensing benefit across all spectrums of the global population.

## 1. Introduction

### 1.1. Epidemiology of Broccoli and Health

The collective retrospective (observational), prospective, and interventional evidence for the beneficial effects of broccoli on health is robust. The former two categories will be briefly summarized herein, and the latter make up the bulk of this review. Broccoli was thought to have been domesticated in the 1500s, brought to the UK in the early 1700s, and to the future United States in the late 1700s, but was little-known in the USA until the 1920s [1]. Thus, as a relatively new crop, one can only point to about a century of relatively widespread consumption. The epidemiology of broccoli′s impact on health covers precisely half of that century, beginning with Graham′s early work showing a dose-response relationship between the consumption of broccoli and other cruciferous vegetables, on colon cancer [2]. Since then there have been impressive demonstrations of risk reduction associated with cruciferous vegetables and/or broccoli for bladder cancer [3] and prostate cancer [4] just to name a few. We and others have recently reviewed the growing body of epidemiologic and mechanistic work implicating cruciferous vegetables in general, broccoli specifically, and sulforaphane, with respect to its association with neurologic, neoplastic, dermatologic, and other conditions (e.g., [5,6,7,8,9]). Organizations including the World Cancer Research Fund and the American Institute for Cancer Research have chosen to highlight lifestyles (a Mediterranean type diet) and food groups (non-starchy vegetables or fruit) as featuring strongly in cancer prevention, and they have moved away from recommending specific fruits or vegetables vis à vis cancer risk [10]. However, all evidence points to broccoli and more specifically to sulforaphane from broccoli, and its biogenic precursor glucoraphanin, as being protective against a variety of chronic, and even infectious (e.g., *Helicobacter pylori*) conditions [11].

### 1.2. Discovery of Sulforaphane as a Bioactive Isothiocyanate

Sulforaphane was described in the middle of the last century, as an antibiotic, and was isolated from red cabbage, and from the western USA rangeland weed hoary cress [12]. Various groups have since synthesized it, but Talalay and Zhang were the first to isolate it from broccoli [13] and to demonstrate its cancer protective properties [14]. Its biogenic precursor, glucoraphanin, was then found in abundance in broccoli sprouts and sulforaphane was confirmed to be active in animal carcinogenesis models [15]. Structure-activity evaluation of a series of over 100 synthetic analogs made by Posner and colleagues did not find a more potent inducer of cytoprotective (Phase 2) enzymes than sulforaphane [16], and indeedeme sulforaphane remains one of, if not, the most potent naturally occurring inducers yet discovered [17]. We and many others have identified subsequently multiple pathways and metabolic consequences of this molecule’s presence in mammalian cells, tissues, and in the human body [8,18,19]. While far too numerous to review herein, we note this multiplicity of effects has been well reviewed by others [20,21,22]. Our early work showed that in the undamaged plant there is very little if any free sulforaphane, all of it being present in the form of its biologically inactive precursor glucoraphanin [15]. Thus, it is important to consider the factors controlling glucoraphanin biosynthesis in plants.

### 1.3. Biosynthesis and Function of Glucoraphanin in Broccoli

**Scheme 1:**
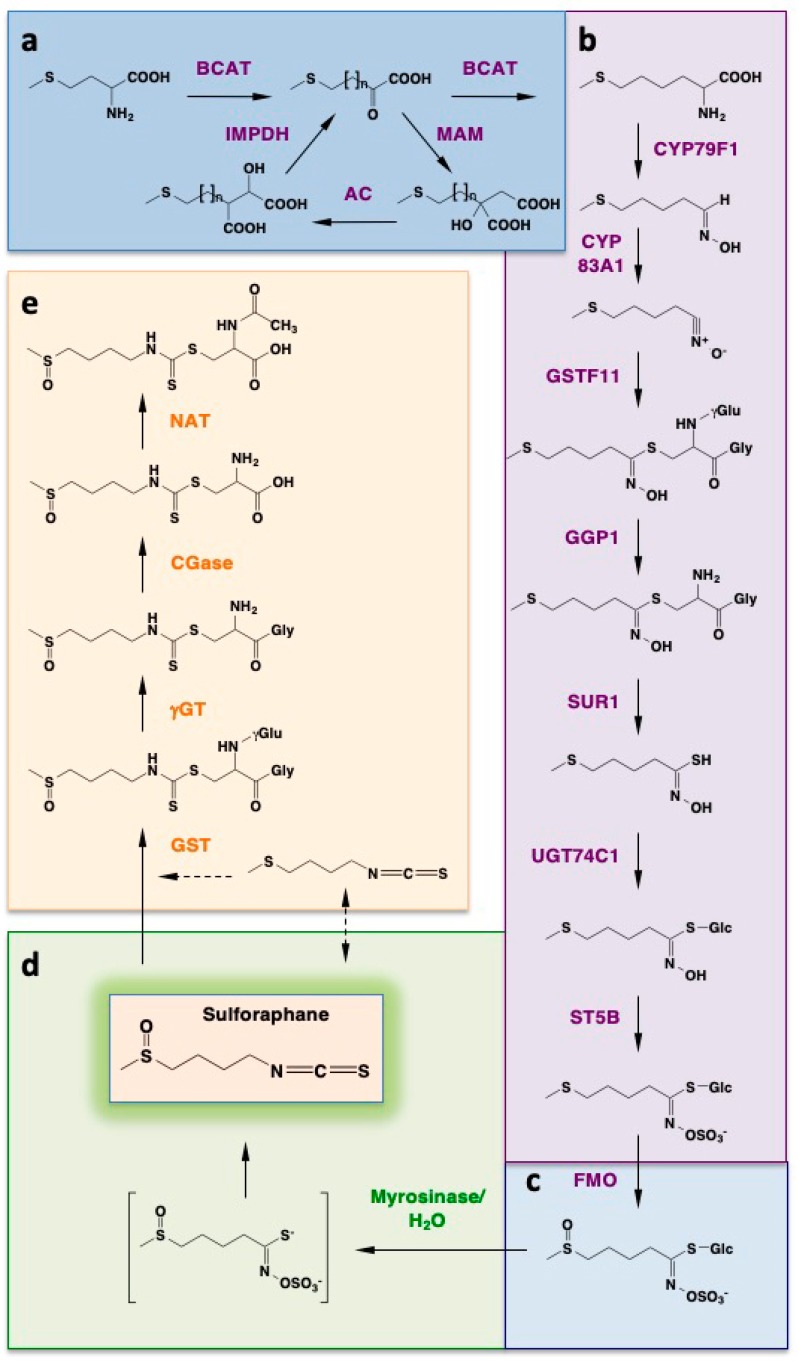
Biosynthesis of glucoraphanin, its hydrolysis to form the isothiocyanate sulforaphane, and metabolism of sulforaphane. The highly reactive isothiocyanate sulforaphane is produced in plants as an inert precursor, the glucosinolate glucoraphanin. Its biosynthetic pathway originates from the amino acid methionine and proceeds in three stages: (i) methionine side chain elongation by two methylene groups (**a**); (ii) formation of the core glucosinolate structure (**b**); (iii) secondary modification of the glucosinolate side chain (**c**); Upon disruption of the plant tissue integrity, glucoraphanin comes into contact with myrosinase, which catalyzes the hydrolysis of glucoraphanin to give sulforaphane (**d**); In mammalian cells, sulforaphane is metabolized through the mercapturic acid pathway, and can also undergo an interconversion to erucin (**e**).

The glucosinolate glucoraphanin is derived from the amino acid methionine. The biosynthetic pathway leading to glucoraphanin formation *in natura* is elaborate and includes three separate stages (Scheme 1) [23,24]. The first stage results in the elongation of the methionine side chain by two methylene groups. The second stage forms the core glucosinolate structure. The third stage constitutes a secondary modification of the glucosinolate side chain.

During the first stage (Scheme 1a), a cytoplasmic branched chain amino acid aminotransferase (BCAT) catalyzes the transamination of methionine to give 2-oxo-4-methylthiobutanoic acid. In turn, this α-keto acid is elongated by two methylene groups in a cyclical mechanism which involves two rounds of three successive transformations that take place in the chloroplast: (i) a condensation with acetyl-CoA, which is catalyzed by a methylthioalkyl malate synthase (MAM); (ii) an isomerization catalyzed by aconitase (AC); (iii) an oxidative decarboxylation catalyzed by an isopropylmalate dehydrogenase (IPMDH). The final product of these transformations, 2-oxo-6-methylthiohexanoic acid, is converted to dihomomethionine in a transamination reaction catalyzed by a BCAT.

During the second stage (Scheme 1b), dihomomethionine undergoes a cytochrome P450 (CYP)-mediated conversion to an aldoxime, which is then further oxidized to a nitrile oxide and subsequently conjugated to a sulfur donor, such as glutathione (GSH). The conjugation reaction can occur non-enzymatically or can be catalyzed by a glutathione S-transferase. The resulting S-alkyl-thiohydroxymate is converted to a thiohydroxymate in a reaction catalyzed by the C-S lyase SUR1.

Because this enzyme requires a free amino group within the substrate, an intermediate step is required: The hydrolytic removal of the γ-glutamyl residue within the conjugate between glutathione and the activated aldoxime, a reaction catalyzed by γ-glutamyl peptidase (GGP). The last steps of the second stage are a glucosyltransferase-mediated S-glucosylation resulting is the formation of a desulfoglucosinolate, followed by sulfation catalyzed by a sulfotransferase (ST). Thus, the parent glucosinolate, 4-methylthiobutyl glucosinolate (glucoerucin) is produced.

During the final stage (Scheme 1c), a secondary modification of the glucosinolate side chain occurs. This is accomplished by an S-oxygenation reaction, which is catalyzed by a flavin monooxygenase (FMO). Together, these elaborate biotransformations, which involve 13 enzymes, result in the synthesis of glucoraphanin. Notably, in *Arabidopsis* the biosynthesis of glucosinolates is regulated by light (being downregulated in prolonged periods of darkness and greatly increased following exposure to light) and shows diurnal variations that are coordinated with those of general sulfur metabolism [25].

Glucoraphanin is chemically stable and biologically inert. However, following plant tissue injury, such as biting or chewing, glucoraphanin comes in contact with the enzyme myrosinase, a ß-thioglucosidase, which in the intact plant is physically separated from its substrate. Myrosinase catalyzes the hydrolysis of glucoraphanin to liberate glucose and form an unstable aglucone (Scheme 1d) that spontaneously rearranges to give rise to a range of products, the most reactive of which is the isothiocyanate sulforaphane. Importantly, mammalian cells do not produce myrosinases; however, the conversion of glucoraphanin to sulforaphane still occurs in mammals. It is carried out by the bacterial microflora of the gastrointestinal tract and can be greatly reduced by antibiotic treatment or mechanical bowel cleansing [26]. Of note, this microbially-mediated conversion of glucoraphanin has been exploited in a recent study to generate high concentrations of sulforaphane locally in the colon of mice [27].

In plants, the main function of the glucosinolates is considered to be defense against pathogens and herbivores, and this has largely been attributed to the isothiocyanate hydrolytic products. Resistance to pathogens positively correlates with the glucosinolate content of the plant [28], which also affects the fungal species composition in the soil [29]. In addition, there is a correlation between alterations in the glucosinolate profile of the plant and basic physiological processes, such as photosynthesis and growth, that occur during abiotic stress, including drought, extreme temperatures, light, salinity, and nutrient deprivation [30]. Taken together, these correlations suggest that the glucosinolates function for protection of the plant against a wide array of environmental challenges. It is therefore not surprising that domesticated lines of Brassica oleracea have lower glucosinolate levels compared to wild species [31]; conversely, the glucoraphanin content of certain wild Brassica species (B. villosa) is high, and this species has been used to generate broccoli hybrids with enhanced concentrations of glucoraphanin [32]. In addition to their role in plant defense, high levels of isothiocyanates have been shown to reduce the biomass of the plant, and interestingly, mutants deficient in glutathione biosynthesis are more susceptible than their wild-type counterparts to the growth-inhibitory effect sulforaphane [33], suggesting similarities in sulforaphane metabolism in plants and mammals.

### 1.4. Glucoraphanin Levels in Broccoli

**Figure 1:**
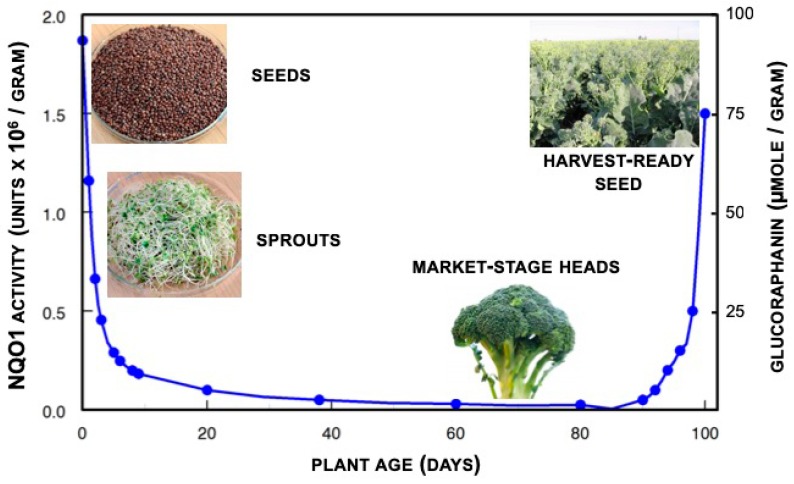
On a weight basis, glucoraphanin (*right axis*) is most abundant in the seeds of the broccoli plant. Upon enzymatic conversion to sulforaphane, the capacity of extracts of these plants to induce or up-regulate phase 2 enzymes such as NQO1 in mammalian cells, follows precisely the same curve (*left axis*).

Glucoraphanin occurs in all tissues of broccoli plants, though it is most abundant in the aerial portions and the developing florets (flower buds) and ultimately the seeds, are richest in this compound (Figure 1). Although two or three other edible cruciferous (*Brassica*) species contain significant amounts of glucoraphanin, it is not as widespread as popular culture would have it. In one sampling of 31 fresh, uncooked broccoli (*Brassica oleracea* var. *italica*) heads, each from different Baltimore area supermarkets, we found a mean of 0.38 μmol glucoraphanin per gram fresh weight, but with a range of from less than 0.005 to 1.13 μmol/gram [34]. Levels of glucoraphanin when tested in over 75 different genotypes of field-grown hybrid broccoli averaged 0.88 and 1.10 μmol glucoraphanin per gram fresh weight when grown in the same fields in two consecutive years [35]. Further work with 32 different genotypes grown across three different years, in the field and the greenhouse produced a similar range of values and a mean glucoraphanin content in broccoli heads of 0.36 μmol per gram fresh weight [36]. Glucoraphanin level in broccoli seeds was judged to be largely determined by genotype, though the environment in which the plants are grown (e.g., location, year, drought, pollution, and disease pressure) also plays a clear and significant role [37].

## 2. Broccoli-Based Intervention in Rodents

### 2.1. Formulation, Route of Administration and Dose

We surveyed the now vast literature on the evaluation of sulforaphane as an agent for disease prevention in mouse and rat models. While some studies reported feeding animals with broccoli (typically lyophilized) incorporated into rodent diets, the large majority have examined efficacy (monitored through molecular, biochemical, biological or pathological endpoints) of sulforaphane as a discrete, commercially available research-grade chemical. Of note, while *R*-sulforaphane is found naturally, the synthetically derived *R,S*-sulforaphane has been likely used in most animal studies due to historical matters of availability and relative cost. Most publications do not directly account for the form of sulforaphane used. There is some suggestion that *R*-sulforaphane shows increased effectiveness compared to its racemic *R,S* counterpart in various models. This review focuses on animal studies using sulforaphane as the test article as few of the dietary studies with broccoli-based preparations attempted to accurately determine the administered bioactive dose.

**Figure 2:**
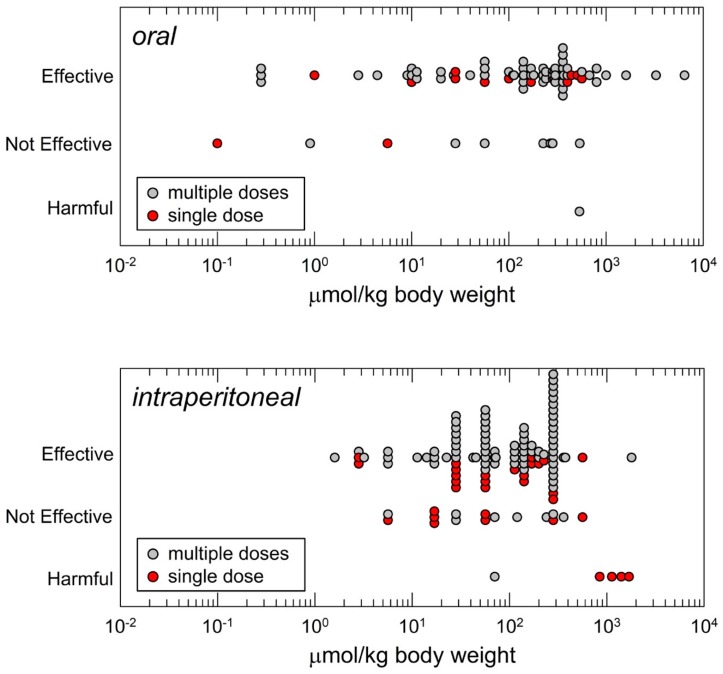
Distribution of daily doses of sulforaphane administered to mice as reported in the literature based on route of administration and efficacy outcome. *Top panel*, oral (gavage or in diet); *bottom panel*, intraperitoneal administration. Where necessary, dose extrapolations assumed 25 g body weight and dietary intake of 4 g food/mouse/day [38].

Animal studies have principally used three routes of administration for sulforaphane: Oral, intraperitoneal and topical. Figure 2 highlights the distributions of doses selected by investigators for oral or intraperitoneal dosing to mice. Oral administration is the route typically used by the NCI in chemopreventive agent development [39]. Yet, somewhat paradoxically as depicted in Figure 2, intraperitoneal administration has been the most commonly employed route for studies with sulforaphane. Presumably this choice reflects relative ease of administration to animals rather than attempted mimicry of a route most appropriate for administration of a dietary compound or matrix to humans.

That said, dose ranges for intraperitoneal administration appears to provide roughly equivalent pharmacological efficacy to oral dosing with sulforaphane, presumably reflecting the excellent bioavailability of this agent. Doses selected for oral administration have spanned a greater than 4-log range and those for intraperitoneal administration an overlapping 3-log range. The median effective dose of sulforaphane in the published literature by oral administration is 175 µmol/kg body weight and by intraperitoneal administration is 113 µmol/kg. Likely reflecting publication bias, most studies irrespective of route of administration report a positive outcome on efficacy. Nonetheless, also informative, is the observation that studies, albeit far fewer in number, report a lack of efficacy in some models: E.g., tumor xenografts or chemical carcinogenesis, bacterial infections, airways inflammation and cerebral ischemia. For most of the listed “not effective” responses, significant responses were reported for study endpoints at higher doses as part of dose-response evaluations. Very few studies report harmful outcomes but are notable as discussed later. Topical application of sulforaphane has been used effectively in limited settings for mitigation of skin erythema, inflammation or photocarcinogenesis.

The literature surveyed is listed and annotated for studies employing oral (Table S1), intraperitoneal (Table S2), and topical (Table S3) administration of sulforaphane to mice as well as oral administration to rats (Table S4).

### 2.2. Efficacy Endpoints: Mechanisms Versus Dose, Risks and Benefits

**Figure 3:**
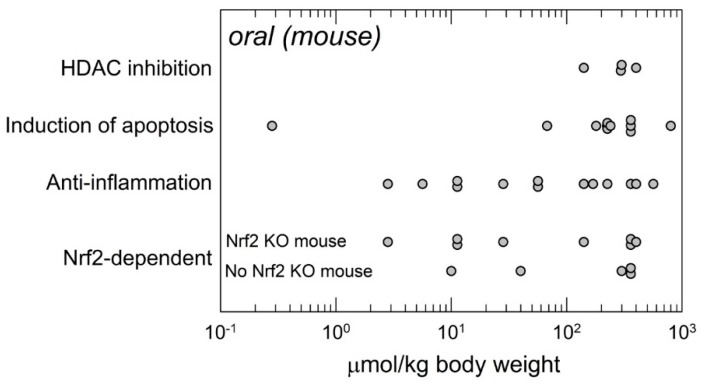
Distribution of oral doses of sulforaphane administered to mice in studies that included experimental examination of underlying mechanisms in vivo. Data are as reported and interpreted in the original publications. Listed mechanisms are not necessarily exclusive. Nrf2 KO: Nrf2 knockout. Some studies included comparisons of responses in wild-type and Nrf2 KO mice to impute Nrf2-dependence and are also included in the listed mechanisms (primarily “anti-inflammation”).

Relatively few studies have rigorously examined the mechanisms of action in vivo of sulforaphane that underlie the reported biological outcomes (Figure 3). This experimental paucity lies in stark contrast to the multitude of studies reporting on mechanistic actions identified in cell culture systems as reviewed elsewhere [40,41]. A recent bibliometric review indicates that sulforaphane is the most cited natural product activator of Nrf2 signaling [42]. Indeed, several studies have compared the action of sulforaphane on Nrf2 signaling and disease prevention by comparing outcomes in wild-type and Nrf2-knockout mice. In several cases, Nrf2-dependent effects have been linked to anti-inflammatory actions. In both settings (with some overlapping studies), a wide range of doses have been employed, indicating at least lower bounds for efficacy (3–10 µmol/kg). Studies examining the induction of apoptosis in vivo, principally in xenograft anti-tumorigenesis experiments have utilized high doses (>60 µmol/kg). A similar reliance on higher doses is reflected in studies on the inhibition of histone deacetylase (HDAC) activities (>100 µmol/kg). Absence of data in these cases should be distinguished from absence of effect at lower doses. Further experiments are required. To this point, relatively few dose-response studies have been conducted in vivo, so it is not possible to appreciate whether a plot of the relationship of response(s) to sulforaphane dose may take the form of “S”, “∩” or possibly some other shape. No maximally effective doses have been established—They are likely to vary depending upon the endpoint.

Several studies conducted with higher doses of sulforaphane in mice do describe toxicities that require careful attention to risk-benefit analyses and determination of therapeutic or prophylactic indices. Socala et al. [43] examined the toxicity profile of sulforaphane in mice after intraperitoneal injection of single doses. High doses of sulforaphane produced marked sedation (at 150–300 mg/kg), hypothermia (at 150–300 mg/kg), impairment of motor coordination (at 200–300 mg/kg), decrease in skeletal muscle strength (at 250–300 mg/kg), and deaths (at 200–300 mg/kg). The LD_50_ value of sulforaphane in mice was estimated to be 213 mg/kg i.p. (1203 µmol/kg). This value is about 10-fold higher than the median dose reported for efficacy outcomes in mice. Sulforaphane (at 100 mg/kg) potentiated the anticonvulsant efficacy of carbamazepine in a seizure test. This drug interaction could have been pharmacokinetic in nature, a form of interaction also considered but not observed in humans [44]. Shorey et al. [45] observed increased morbidity and no reduction in lung tumorigenesis in offspring born to mothers receiving transplacental and lactational exposure to the carcinogen dibenzo[*def,p*]chrysene and supplemented with dietary sulforaphane (400 ppm) or its primary whole food source, broccoli sprouts (10% *w*t/*w*t), contrasting to many reports of chemoprotection in adult animal models. Of potentially greatest concern is the study of Tao et al. [46]. They utilized a chemical carcinogenesis model with vinyl carbamate (A/J mice) and a genetic model (LSL-K-rasG12D/+ mice) to induce lung cancers. Mice were treated intraperitoneally with 12.5 mg/kg (75.5 µmol/kg) (5 doses, once every three days during tumor induction (pre-treatment) or 12.5 mg/kg every three days, starting one week after tumor induction for 13 weeks (post-treatment). In the chemical carcinogenesis model, pre-treatment reduced the number of tumors, and post-treatment slightly promoted tumors. In the genetic model, pre-treatment with SF had no effect on tumor number, but post-treatment increased tumor number and size. Given observed efficacy in humans at doses <0.5 µmol/kg of sulforaphane, and providing allowance for allometric scaling from mice and the improved bioavailability with i.p. dosing, there is only a 10-fold difference in safety margin. It should be noted that Kombairaju et al. [47] reported that prolonged sulforaphane treatment (0.5 mg, 5d/wk for 3 mo. by means of a nebulizer) did not enhance tumorigenesis in the same LSL-K-rasG12D/+ mouse model. In settings of long-term preventive interventions with sulforaphane or a broccoli-based formulation, careful consideration will need to be given to matters of dose, schedule and duration.

## 3. Broccoli-Based Clinical Trials

### 3.1. Sulforaphane Pharmacokinetics and Pharmacodynamics

Upon entry into the mammalian cell, sulforaphane is conjugated with GSH in a GST-catalyzed reaction, entering the mercapturic acid pathway (Scheme 1e). The glutathione conjugate of sulforaphane is subjected to a series of sequential conversions catalyzed by γ-glutamyltranspeptidase (γGT), followed by cysteinylglycinase (CGase), and N-acetyltransferase (NAT). The final product is the *N*-acetylcysteine conjugate of sulforaphane (mercapturic acid). In addition, sulforaphane can undergo an interconversion to erucin [1-isothiocyanato-4-(methylthio)butane], which is then metabolized in an identical manner to that for sulforaphane [48,49].

Sulforaphane and its metabolites (dithiocarbamates) can be quantified collectively by cyclocondensation with 1,2-benzenedithiol, with sensitivity in the picomolar range [50]. This highly sensitive, simple and convenient method has been widely used to measure the levels of sulforaphane and its metabolites in blood, plasma, urine and tissues following sulforaphane administration to rodents and humans. The use of this method revealed that sulforaphane crosses the placental barrier based on detection of dithiocarbamates in embryos 2 h post-treatment of pregnant mice with a single (5 μmol) dose of sulforaphane [51]. In addition, methods have been developed to analyze the individual metabolites following their separation by liquid chromatography coupled with tandem mass spectrometry (LC-MS/MS) [52,53]. Furthermore, the use of mass spectrometry coupled with stable isotope-labeled internal standards of sulforaphane [1-isothiocyanato-4-methyl-sulfinyl(1,1,2,2,3,3,4,4-^2^*H*8)butane] and its corresponding mercapturic acid pathway conjugates allows for quantitative, precise, sensitive, and specific analysis of sulforaphane and its metabolites [54].

With these analytical tools in hand, a number of pharmacokinetic studies have been conducted in rodents and humans. Thus, following oral administration of an exceedingly high dose of 150 μmol sulforaphane to 10-week-old female Sprague–Dawley rats, the concentration of dithiocarbamates in the plasma of the animals increased rapidly reaching a peak (*C*_max_) of 60 μM 1 h after dosing, with area under the concentration curve (AUC) of 491 h μmol/L, elimination constant (*K*_el_) of 0.1 h^−1^, and elimination half-life of 6.7 h [55]. Similarly, following oral administration of 200 μmol broccoli sprout isothiocyanates to four healthy human volunteers, the peak plasma dithiocarbamate concentration (*C*_max_) was 1.91 ± 0.24 (0.943–2.27) μM 1 h after dosing, with half-life of 1.77 ± 0.13 h, and clearance of 369 ± 53 mL/min [56]. A study in 20 participants administered 200 μmol sulforaphane as sulforaphane-rich powder in capsules reported a *C*_max_ of 0.7 ± 0.2 µM at 3 h, with a half-life of 1.9 ± 0.4 h for elimination of sulforaphane equivalents measured by mass spectrometry [57]. Another pharmacokinetic study, in which a single dose of broccoli soup delivering the equivalent of either 16 μmol or 52 μmol of sulforaphane was administered, reported *C*_max_ of 2.2 ± 0.8 µM and 7.3 ± 2.9 µM at 1.5 h and 2 h for the low and the high dose, respectively [53]. A double-blinded, randomized crossover trial with broccoli soups (prepared from plants with increased glucoraphanin content) delivering a single dose of 84, 280, or 452 μmol of glucoraphanin documented peak plasma concentrations (*C*_max_) of 0.17 ± 0.12, 0.37 ± 0.26, and 0.61 ± 0.40 μM, respectively [52]. Another study reported plasma dithiocarbamate levels of 0.92 ± 0.72 μM and mean epithelial-/stromal-enriched breast tissue dithiocarbamate concentration of 1.45 ± 1.12 and 2.00 ± 1.95 pmol/mg tissue for the right and the left breast, respectively in eight healthy women undergoing reduction mammoplasty who had received a single dose of a broccoli sprout preparation delivering 200 μmol sulforaphane 1 h prior to surgery [55].

In a double-blind randomized placebo-controlled trial in men presenting for prostate biopsy, plasma levels of 0.12 µM of sulforaphane and its metabolites were detected after an intervention period of 4–8 weeks with two daily doses of 100 μmol sulforaphane administered 12 h apart [58]. Isothiocyanate levels of 2.2 µM and 500 nM were detected in the plasma and synovial fluid, respectively of patients with osteoarthritis undergoing knee replacement surgery following consumption of glucosinolate-rich diets for 2 weeks [59].

A study in healthy subjects who received single oral doses of broccoli sprout extracts containing the equivalent of 111 µmol of glucosinolates or isothiocyanates showed cumulative urinary dithiocarbamate excretion of 88.9 ± 5.5 μmol and 13.1 ± 1.9 μmol for the isothiocyanate and the glucosinolate preparation, respectively [60]. This study further revealed that for the isothiocyanate preparation, excretion was consistent and linear over a 25–200 μmol dose range, whereas for the glucosinolate preparation, excretion was highly variable among individuals. These observations are in close agreement with results from a randomized, placebo-controlled, double-blind Phase I clinical trial, in which isothiocyanate (25 μmol)- or glucosinolate (25 μmol or 100 μmol)-rich preparations were orally administered to three cohorts of three healthy human subjects at 8-h intervals for 7 days; one other subject in each cohort received placebo [61]. Notably, this study showed no evidence of clinically significant adverse events based on 32 types of hematology and chemistry tests, including liver (transaminases) and thyroid (TSH, T3, and T4) function tests. In agreement, a recent analysis of biochemical parameters of thyroid function in serum collected from 45 female volunteers that had participated in a randomized clinical trial revealed no alterations compared to baseline following an intervention for 12 weeks with a broccoli sprout beverage containing a combination of 40 μmol sulforaphane and 600 μmol of glucoraphanin [62].

The finding that compared to isothiocyanates, oral administration of glucosinolates results in lower bioavailability, slower elimination, and greater inter-individual variation in excretion was further strengthened by a larger (50 participants) crossover clinical trial that involved 5-day baseline period followed by daily administration of broccoli sprout beverages delivering either glucosinolates or their corresponding isothiocyanates for 7 days, 5-day washout period, and 7-day administration of the opposite intervention [63]. Using fecal sample collections from five subjects with high 24-h urinary excretion profiles (‘high converters’) and five subjects with low excretion profiles (‘low converters’), it was found that ex vivo, the degradation of glucoraphanin was greater in cultures of fecal bacteria derived from the ‘high converters’ in comparison to the ‘low converters’ [64]. These observations are consistent with earlier work showing that mechanical cleansing or antibiotic treatment greatly reduce the glucosinolate conversion in healthy human subjects [26] and indicate that the gastrointestinal microflora represents a critical factor in determining the extent of gl ucosinolate hydrolysis. In addition to the inter-individual variations, there are also diurnal variations in the conversion of glucosinolates to dithiocarbamates, whereby conversion is greater during the day [65]. By contrast, the conversion of isothiocyanates to dithiocarbamates is higher during the night.

Overall, in humans, sulforaphane is rapidly absorbed and eliminated with small inter-individual variations and typical urinary excretion of 70% to 90% of the dose. By contrast, the conversion of glucoraphanin is slow and with high inter-individual variations. The urinary excretion of sulforaphane metabolites following intervention with glucoraphanin-containing preparations typically range from 2% to 15% of the dose, being 1% to 45% at the extremes. The differences in inter-individual variations between sulforaphane and glucoraphanin make, at first glance, the use of sulforaphane much more attractive for the purposes of dose precision. However, in contrast to its stable glucosinolate precursor, sulforaphane is unstable, which has prompted the development of stabilized preparations, such as an α-cyclodextrin-encapsulated form of sulforaphane [66] and a stabilized version of pure plant-derived sulforaphane, known as Prostaphane^®^ (Nutrinov, Noyal sur Vilaine Cedex, France). Alternatively, glucoraphanin-rich preparations containing active myrosinase have also been used [67,68]. As formulations differ in their bioavailability (which provides a possible explanation for the differences in pharmacokinetic parameters reported in the various human studies), the excreted amount of sulforaphane metabolites in the urine, and not the amount in the administered preparation, provides a more reliable measure of the actual dose [69].

Similar to the studies of the pharmacokinetics of sulforaphane, nearly all human studies addressing the pharmacodynamics of sulforaphane have used glucoraphanin- or sulforaphane-rich broccoli-based preparations. Although there is currently no direct evidence for specific target engagement by sulforaphane in humans, there is clear evidence for its pharmacodynamic action. Thus, increased levels of the Nrf2-target enzymes A-class GSTs and NQO1 have been reported in plasma [70] and saliva [71] of human subjects consuming cruciferous vegetables. In agreement, administration of glucoraphanin/sulforaphane-rich preparations to healthy volunteers resulted in increased mRNA or protein levels of NQO1 and GSTs in PBMC, skin punch biopsies, as well as in nasal and buccal scrapings [72,73,74,75,76].

Broccoli-based glucoraphanin/sulforaphane-rich preparations have been shown to accelerate the detoxication and excretion of potentially carcinogenetic food contaminants and air pollutants, offering a very attractive strategy for population-wide reduction in cancer risk due to unavoidable exposures to pollution. A cross-over clinical trial with 50 human volunteers, which was conducted in Qidong, China, found statistically significant increases of 20–50% in the urinary excretion levels of glutathione-derived conjugates of the air pollutants acrolein and benzene following consumption of sulforaphane- and/or glucoraphanin-rich broccoli sprout-derived beverages [77]. A subsequent 12-week placebo-controlled, randomized clinical trial involving 291 participants from the same area using broccoli sprout beverages containing a combination of 40 μmol sulforaphane and 600 μmol glucoraphanin confirmed and extended these findings by showing that the excretion levels of the glutathione-derived conjugates of benzene and acrolein were significantly increased, by 61% and 23%, respectively in the volunteers who received the broccoli sprout beverage compared with placebo [78]. Very recently, a randomized, placebo-controlled, multidose intervention trial of a broccoli sprout beverage, which was conducted in the same area of China, showed a dose-dependent excretion of the urinary metabolites of sulforaphane, and further found that a treatment regime with daily doses of 40 μmol sulforaphane and 600 μmol glucoraphanin for 10 days, resulting in an urinary excretion of ∼25 μmol sulforaphane metabolites per day, promotes the detoxication of benzene [69].

Global gene expression profiling to evaluate the transcriptional changes in the prostate of men at high risk for prostate cancer has revealed that consumption of broccoli-rich diets for 6- or 12 months associates with transcriptional changes in signaling pathways involved in inflammation and carcinogenesis in the prostate tissue [79,80]; importantly, these changes are dose-dependently attenuated in subjects receiving the glucoraphanin-rich diet [80]. Other pharmacodynamic effects of interventions with glucoraphanin/sulforaphane in humans include: Increase in the levels of reduced glutathione in brain [81], enhanced integration of fatty acid β-oxidation with TCA cycle activity [82], protection against skin erythema caused by exposure to ultraviolet radiation [83,84], reduction in plasma LDL-cholesterol [85], decrease in the levels of fasting blood glucose and glycated hemoglobin in obese patients with dysregulated type 2 diabetes [86], and improvements in social interaction, behavior, and verbal communication in young men with autism spectrum disorder [87]. Overall, although the precise molecular mediators are not always known, it is clear that interventions with glucoraphanin/sulforaphane-rich broccoli preparations in humans lead to diverse beneficial effects.

### 3.2. Clinical Studies with Broccoli-Based Preparations: Efficacy

Broccoli-based preparations consist almost exclusively of either glucoraphanin, sulforaphane, glucoraphanin with added active myrosinase, the raw, cooked, or dried vegetables themselves (either broccoli or broccoli sprouts), or extracts of broccoli seeds or sprouts—glucoraphanin-rich, sulforaphane-rich, or both. These studies are, of course, not all straightforward, and measurement of the broccoli-derived ingredients can be a source of mystery, obfuscation, and confusion, that is readily exploited by the supplement industry. Nonetheless, many clinical studies have now been done, and some overarching learnings can begin to be formulated. We and others have spent much time assessing bioavailability, conversion of glucoraphanin to sulforaphane, safety, and the classic ADME (absorption, distribution, metabolism, and excretion) pharmacokinetic parameters discussed in a previous section of this review. Efficacy has been studied much less thoroughly, but a picture is beginning to emerge. The following areas (in alphabetical order) have received special attention:

#### 3.2.1. Aflatoxin Toxicity 

The levels of aflatoxins from stored, subsistence seeds (corn and peanuts) in the Qidong area of coastal China near Shanghai, have in recent history been exceedingly high, as has associated incidence of liver cancer. Broccoli sprouts have been successfully piloted as a preventive intervention, and much data has been generated on safety and efficacy [biomarkers], as well as tolerance and acceptance by the local population [88,89];

#### 3.2.2. Air Pollution Detoxification 

The natural laboratory in Qidong further served to study the effects of broccoli spouts on detoxication of volatile organic air pollutants [69,77,78,90]. Others, in other environments, have examined effects of diesel exhaust (particulate) and other air pollutants [91,92];

#### 3.2.3. Arthritis

Early work by Healy and collaborators suggested an effect on shear stress in chondrocytes [93]. This has now been followed by the elegant demonstration that increasing broccoli intake results in isothiocyanate (e.g., sulforaphane) uptake into the joint, with concomitant changes in the joint [59]. Much other work on anti-inflammatory properties of sulforaphane may ultimately be related to arthritis;

#### 3.2.4. Asthma and Atopic Allergic Responses

A variety of relatively small but significant effects of sulforaphane have been demonstrated in a number of relatively complex experimental systems [73,92,94,95,96,97];

#### 3.2.5. Cancer Biomarkers

A large variety of biomarkers have now been reported following sulforaphane treatment. The cancers being studied include: Breast [55,98], lung [99]; gastric [100], colo-rectal [101], prostate [79,80,102,103], skin [75,83,84,104], head and neck [72], and liver [90];

#### 3.2.6. COPD

A 90-subject, three-center RCT was conducted with sulforaphane-rich broccoli sprout extract in which no effect was seen on patients with advanced COPD. *Post-facto* reasoning suggested that the already severely degraded condition of subjects’ airways may well not have permitted for responsiveness (Nrf2-related and anti-inflammatory) regardless of the agent used [105]. A follow-up analysis suggests that there was compartmentalization of anti-oxidant and anti-inflammatory gene expression in current and former smokers with COPD [106];

#### 3.2.7. CVD

At least three different groups have reported effects that included biochemical markers [107], blood pressure and flow-mediated dilation [108], and plasma metabolite biomarkers [82,85];

#### 3.2.8. Diabetes, Metabolic Syndrome, and Related Disorders 

Many clinical studies from at least three different groups have now been published. They have shown effects on reduced gluconeogenesis [86], inflammatory markers [109], and insulin resistance [110,111,112];

#### 3.2.9. General

Extensive measures in a variety of studies have documented upregulation of key chemoprotective enzymes. To name only a few studies not mentioned elsewhere in this review: [113,114];

#### 3.2.10. Helicobacter Pylori Infection 

Our initial observations in vitro and in an animal model [11] have been translated to some eradication and more importantly, reduction of the levels of *H. pylori* colonization and gastric inflammation in some infected individuals [115,116,117];

#### 3.2.11. NASH/NAFLD

Positive changes in liver function markers have been observed [118] and much interest continues, based upon encouraging pre-clinical studies;

#### 3.2.12. Neurodegenerative Conditions 

Small studies showing effects on GSH levels and mapping GSH elevations to specific brain regions have generated much excitement [5,81];

#### 3.2.13. Neurodevelopmental Conditions 

One successful and highly visible study showing improvement of conditions of autism spectrum disorder (ASD) following consumption of sulforaphane from broccoli sprouts [87,119] is now being followed up with at least five studies with ASD subjects, two with schizophrenia, and one with psychoses. Results of only one of these follow-on studies has published so far [120];

#### 3.2.14. Sickle Cell Disease

Although only a single, small, dose escalation Phase 1 trial has been run, a limited number of disease-relevant pharmacodynamic endpoints were queried, and safety was evaluated through a relatively high calculated dose [74].

### 3.3. Summary of Clinical Studies with Sulforaphane, Glucoraphanin or Mixtures

Table 1 summarizes the published literature of clinical trials conducted with broccoli-based preparations, sulforaphane and/or glucoraphanin. Also included are clinical trials listed on ClinicalTrials.gov that are known to be underway. Pending trials are not included. Oral doses that delivered sulforaphane directly ranged from 9.9 to 847 µmol per person per day (median 100 µmol) and for glucoraphanin 25 to 800 µmol per person per day (median 190 µmol). The trend towards the use of higher doses of glucoraphanin versus sulforaphane reflect considerations on the part of study directors on the limited conversion of glucoraphanin to sulforaphane in the absence of exogenous myrosinase in beverage or dietary supplement preparations.

The harsh taste (a.k.a. back-of-the-throat burning sensation) that is noticed by most people who consume higher doses of sulforaphane, must be acknowledged and anticipated by investigators. They must make accommodations for what some subjects may consider a highly objectionable taste. This is particularly so at the higher limits of dosing with sulforaphane, and not so much of a concern when dosing with glucoraphanin, or even with glucoraphanin-plus-myrosinase. It is this harsh or burning sensation that has led in part to the characterization of the glucosinolate-myrosinase-isothiocyanate system as “the mustard oil bomb” [121]. This should not, however, be confused with the bitter descriptor leveled at most brassica vegetables and moringa, that appears to have more to do with the TAS2R class of taste receptors.

The presence and/or enzymatic production of levels of sulforaphane in oral doses ranging above about 100 µmol, creates a burning taste that most consumers notice in the back of their throats rather than on the tongue. This prevents some from consuming broccoli/sulforaphane preparations. Higher doses of sulforaphane lead to an increased number of adverse event reports, primarily nausea, heartburn, or other gastrointestinal discomfort [44,67,87,119,122]. We have conducted scientifically guided studies to mask or distract consumers from that very distinctive taste, and to facilitate the development of proper placebos [122,123].

## 4. Challenges Ahead

### 4.1. Plants Versus Discrete Isolates: Foods vs. Supplements

As an ultimate public health paradigm, eating plants directly, with or without cooking or minimal processing, must be the solution in most of the world. The wealthiest regions can afford supplements as preventive interventions. Attempting to turn a chemopreventive approach into a pharmaceutical strategy would be foolhardy, but to the extent that sulforaphane is proven to have curative or therapeutic properties, this could be a direction in which to turn. From the perspective of designing and conducting clinical studies to demonstrate preventive efficacy, many issues come to bear on the subject. Though we have dealt with them [65,139,140], certain of them bear repeating:

#### 4.1.1. First

If it is indeed plant foods that will be the ultimate delivery vehicle, then food companies which will share in an enormous upside to successfully enhanced health-span, must bear some of the costs of conducting these trials. Though much has been made of the size and lobbying muscle of “big pharma”, (roughly a trillion dollar industry worldwide), the food sector of the economy in the USA alone is now valued at about $1.1 trillion [141].

#### 4.1.2. Second

Standardization of dose delivery is extremely difficult when conducting clinical studies with foods. As sulforaphane gains scientific credibility as a preventive phytochemical, the clinical studies aiming to determine how, how much, for how long, and how to deliver and measure it, will by definition grow larger, longer, and more sophisticated. Perhaps there will come a time when foods such as broccoli will be labeled with variety name (e.g., like the maddening little stickers on apples) and glucoraphanin titer to enhance the consumer’s ability to make direct health-span purchasing choices. In the meantime, the clinical studies that we have performed with broccoli and broccoli sprouts have already strained the academic system to the breaking point [142]. The food industry needs to step up.

We and others have switched to the use of sulforaphane-containing or sulforaphane-generating supplements as discussed herein. However, the use of commercial supplements is fraught with dangers since there are many poor supplements and many unscrupulous supplement purveyors [143,144]. Robust quality control is essential. Thus, better industry monitoring and regulation, as well as rigorous validation by investigators, of the labeled “doses”, is critical. One cannot for the foreseeable future trust that what appears on the label is an accurate representation of “dose” (and sometimes plant entity), and in Table 1 we have indicated clinical studies in which label results have been used rather than making dose measurements prior to or during intervention.

#### 4.1.3. Third

There must be clinician buy-in to the concept that “thy food is thy medicine, and thy medicine is thy food”. It has been attributed, perhaps fallaciously, to Hippocrates, but regardless of its origin, the physicians who are Hippocrates’ intellectual descendants, have not risen to take the bait. They have instead taken the bait of big pharma, hook-line-and-sinker, and it is threatening to sink our economy. Pharmaceuticals most certainly have their place, but we must recruit the physicians in our society, whom people still trust in matters of their critical and preventive care, to more firmly place at least one foot in the dietary side of the aisle.

### 4.2. Optimizing the Definition of Dose

**Figure 4:**
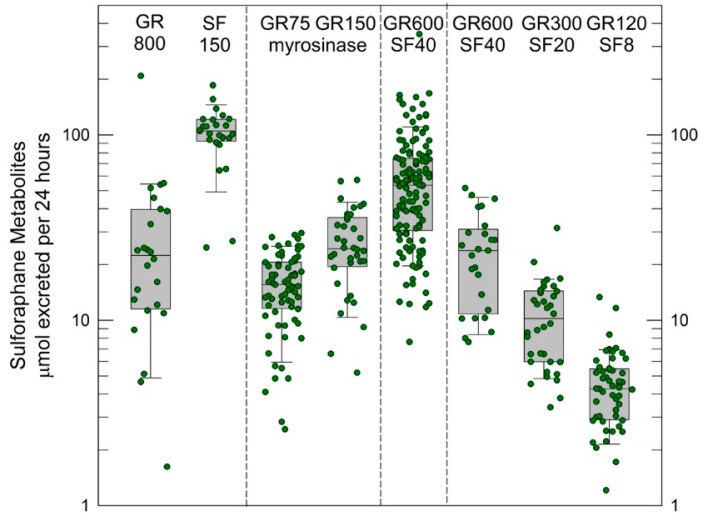
Urinary excretion of sulforaphane metabolites (“internal dose”) per 24 h following an initial dose with different broccoli sprout preparations from intervention studies conducted in Qidong China [63,77,78]. All analyses were conducted by isotope dilution mass spectrometry. Values are the sum of sulforaphane, sulforaphane-cysteine and sulforaphane-*N*-acetylcysteine for each participant. Box plots are median and 5% and 95% confidence intervals. GR 800 was a beverage prepared from a hot-water extract of 3-day old broccoli sprouts that was lyophilized, and then reconstituted in mango juice and water to deliver 800 µmole glucoraphanin (GR); SF 150 was the hot-water extract cooled to room temperature, treated with daikon to deliver myrosinase, then lyophilized and later reconstituted in mango juice and water to deliver 150 µmol of sulforaphane (SF). GR600 + SF 40 were beverages reconstituted in pineapple juice, lime juice and water from their lyophilized powders to deliver a dose of 600 µmol of GR and 40 µmol SF. In addition to beverages, a study was conducted with a commercial dietary supplement formulated as tablets constituted from lyophilized broccoli sprouts and finely milled broccoli seeds to provide glucoraphanin (75 and 150 µmol) in the presence of myrosinase.

Several key challenges in the design of clinical chemoprevention trials, especially food-based trials, are the selection of the dose, formulation, and dose schedule of the intervention material. Dose schedule is typically delineated by the convenience of a daily schedule and knowledge of the short biological half-life of sulforaphane. The other factors are less circumscribed. Selection of dose is complicated by the very different bioavailability of sulforaphane when administered in the precursor form of glucoraphanin and when given as sulforaphane itself, as discussed earlier. Figure 4 summarizes our experiences from four clinical trials conducted in Qidong, China with broccoli-based interventions enriched with glucoraphanin, sulforaphane, or both. 

In a cross-over design study we observed recoveries of excreted sulforaphane metabolites (principally sulforaphane-N-acetylcysteine) to be about 5% when a glucoraphanin-rich beverage was administered with a range of 1 to 45% but about 70% with a much narrow range of inter-individual variation when a sulforaphane-rich beverage was used [68]. Thus, bioavailability changes dramatically as a function of the source material—precursor or bioactive. Some of our subsequent studies used blends of sulforaphane- and glucoraphanin-rich preparations to improve bioavailability together with tolerability and to examine dose-response relationships [69,78]. Higher intake was achieved, but with little impact on inter-individual variability. Formulation, which in turn reflects how broccoli sprout extracts are prepared (e.g., with or without exogenous myrosinase-catalyzed hydrolysis of glucoraphanin), strongly affects bioavailability. Using a dietary supplement formulation of glucoraphanin plus myrosinase (Avmacol^®^) in tablet form, we observed a median 20% bioavailability with greatly dampened inter-individual variability. Fahey et al. [67] have observed approximately 35% bioavailability with this supplement in a different population. Thus, reporting of administered dose of glucoraphanin and/or sulforaphane is a poor measure of the bioavailable/bioactive dose of sulforaphane. As a consequence, we propose that the excreted amount of sulforaphane metabolites (sulforaphane + sulforaphane cysteine-glycine + sulforaphane cysteine + sulforaphane N-acetylcysteine) in urine over 24 h (2–3 half-lives), which is a measure of “internal dose”, provides a more revealing and likely consistent view of the delivery of sulforaphane to study participants. In turn, use of “internal dose” metrics will facilitate optimization of the linkage between formulation, dose, and schedule with determinants of efficacy and, importantly, allow more facile comparisons of results between different clinical trials.

### 4.3. Integrating Animal and Clinical Studies

Epidemiological studies have strongly hinted at beneficial associations between crucifer or more specifically broccoli consumption and mitigation of disease risks. However, it is the animal studies that have provided a robust foundation for the imperative to conduct clinical trials with broccoli, broccoli sprouts and derived-preparations or sulforaphane itself. As summarized in the Supplemental Tables S1–S4, there are a breadth as well as depth of studies describing roles for sulforaphane in the prevention and treatment of diseases in mouse and rat models. 

**Figure 5:**
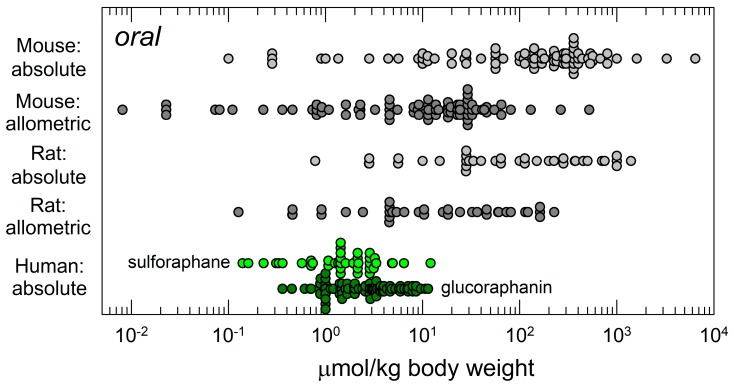
Comparisons of published oral doses of sulforaphane administered to mice or rats and sulforaphane (tablets or sulforaphane-rich broccoli preparations) or glucoraphanin-rich broccoli preparations administered to humans. The allometric scaling of the murine doses uses the correction factor of 0.081 and those for rat doses 0.162 [145]. Human doses were based on an estimate of 70 kg body weights in each study.

Yet, animal studies have not delivered all that might be expected of them. It is clear from the data presented in Figure 5 that the pre-clinical experimentalists have not thought carefully about the selection of dose (or route) and its relevance to clinical utility. Using oral dosing in mice and rat studies as examples, over two-thirds of the animal studies have used doses that exceed the highest (and bordering on intolerable) doses of sulforaphane used in humans, even after accounting for allometric scaling between rodents and humans. Few studies have included a dose-response; thus, the greater than 4-log spread of doses used in mice appears to be driven by needs for effect reporting in publications rather than optimization of translational science. Authors of this review have contributed to this dose skewing, among many investigators.

How were initial doses selected for clinical trials with broccoli-based preparations? Not from any animal studies, which were already in abundance when the first in human studies were conducted. Not from pharmacological studies in rodents to define peak plasma levels, half-lives, metabolic fate or tissue distribution of sulforaphane. Rather, after initial, careful quantitative dose-escalation pharmacokinetic studies by Talalay and colleagues [26,60,61] with crucifers, clinical trialists quickly sought to push doses to the extremes of tolerability by attempting to mask the bitter taste of the simple enriched formulations in order to identify pharmacodynamic action. Only recently have there been attempts to define minimally effective doses in humans—an outcome made possible by the development of consistently formulated, stable, bioavailable broccoli-derived preparations. The disconnect between animal and human studies has been exacerbated because of the public health-based desire (and regulatory-driven need) to conduct the initial human studies with broccoli-based preparations, that in hindsight varied greatly in sulforaphane yield and bioavailability, and the expedient use of pure sulforaphane in most animal studies. Feeding animals lyophilized broccoli or powdered broccoli extracts were likely disfavored because of the need for high percentages within the diet, which could skew overall nutrient uptake. Preparations with stabilized sulforaphane now entering clinical use will close the gap in formulation and could be used to provide more quantitative insights into dose-response [20,102]. Regardless, experimentalists need to now pay close attention to examining efficacy—and underlying mechanisms—using dose and schedule equivalencies to those that are attainable and tolerable in humans—and arguably with oral and not intraperitoneal routes of administration. This notion also extends to in vitro studies, whether in human or animal cell lines, that often report the use of sulforaphane concentrations in far excess of what is likely achievable in human tissues as peak levels, never mind the short biological half-life in vivo that quickly attenuates the duration of the exposure.

### 4.4. Better Efficacy Biomarkers

While there has been substantial progress in the refinement of sourcing and formulation to optimize the pharmacokinetics of broccoli-based preparations now used in clinical trials, there is nonetheless limited information on the factors that contribute to the wide inter-individual variation in pharmacokinetics seen in many clinical trial participants. Host factors (genetic polymorphisms and epigenetics) as well as host-microbiome interactions likely play important roles in this variability. Additionally, there is a striking need to develop rigorous biomarkers of pharmacodynamic action. Better links between purported mechanisms of action delineated in the pre-clinical settings and functional assessments of clinical efficacy are needed to optimize interventions and to better identify those healthy or at-risk groups that might best benefit. With more than 50 papers published on clinical studies with broccoli, glucoraphanin-rich and/or sulforaphane-rich broccoli beverages, powders and dietary supplements, as well as stabilized formulations of sulforaphane, there is ample optimism for disease preventing or mitigating applications to continue evolving. The additional listings of more than 50 proposed/ongoing trials in ClinicalTrials.gov also bears witness to such expectations. Short-term markers of pharmacodynamic actions—likely supported by -omics platforms - are needed to serve as guideposts for rapid deployment of existing and emerging knowledge for the betterment of health using broccoli-based strategies.

## Figures and Tables

**Table 1:** Summary of Clinical Trials with Broccoli, Glucoraphanin or Sulforaphane.

			Compound Delivered^2^			Delivery Format^3^			Non-Fresh Product Source^4^							
Year^1^	First Author	Study Population	SF	GR	GR+ Myr	Tabs/Caps	Powder/Other	Fresh	Commercial	Academic	Treatment^5^	Sample No	Dose as Reported, Converted to μmol^6^	μmol/kg BW^7^	Results	Ref
1998	Shapiro	Healthy	**x**	**x**	**x**			**x**			250 g fresh broccoli (180 μmol GR) or 144 μmol GS or 118 μmol isothiocyanates (ITC)	6	180 μmol GR	2.57 (GR)	PK following GR or GS or ITC ingestion: Reproducibility and dose-dependence as measured in urine using the cyclocondensation	[26]
2000	Conaway	Healthy		**x**	**x**			**x**			200 g fresh or steamed broccoli w/ 220 and 200 μmol ITCs respectively	12	220 & 200 μmol of total ITCs (measured and assumed to be primarily SF)	3.14/2.86 (SF)	Bioavailability of ITCs from fresh broccoli is greater than that from cooked broccoli	[124]
2002	Ye	Healthy	**x**				**x**			**x**	200 μmol SF	4	broccoli extracts containing predominantly SF; 200 μmol total ITC	2.86 (SF)	Development of a sensitive and specific method for quantifying levels of ITC and their metabolites in human plasma, serum, and erythrocytes	[56]
2004	Murashima	Healthy			**x**			**x**			100 g fresh broccoli sprouts/d x 7 d	12	we calculate a maximum of 500 μmol GR/d	7.1 (GR)	Improvement of lipid metabolism; HDL cholesterol increased significantly only among females	[107]
2004	Walters	Healthy		**x**				**x**			250 g broccoli or brussels sprouts (cooked), per day for 12 days	20	(est.) ca. 25 μmol GR	0.36 (GR)	Induced metabolism of PhIP in humans	[101]
2005	Gasper	Healthy		**x**				**x**			100 g florets from standard broccoli and high-glucosinolate broccoli, one dose	16	150 mL of 107.5 or 345.8 μM “sulforaphane metabolites” broccoli soup	0.23 or 0.74 (SF)	GSTM1 genotypes have significant effect on the SF metabolism	[53]
2005	Kensler	Healthy		**x**				**x**		**x**	broccoli sprout extract (BSE) containing 400 umol or <3 umol GR (placebo), daily for 2 weeks.	200	<3 μmol GR/day or 400 μmol GR/day	0.043/5.71	Decreased urinary excretion of dithiocarbamates and aflatoxin-DNA adducts and trans, anti-phenanthrene tetraol in urine by broccoli sprout glucosinolates.	[90]
2004	Galan	*Helicobacter pylori* infected adults	**x**	**x**	**x**			**x**			14, 28, or 56 g broccoli sprouts 2x/d x 7 days	7	up to 280 μmol GR/day	1, 2, 4 (GR)	7 of 9 patients were stool antigen negative immediately after the completion of therapy and six remained negative at day 35.	[115]
2006	Shapiro	Healthy	**x**	**x**						**x**	BSE containing 25 μmol GR, 100 μmol GR, or 25 μmol SF, 3x/d x 7 d	12	75 & 300 μmol GR/d; 75 μmol SF/d	1.97 & 4.29 (GR); 1.07 (SF)	Cumulative excretion of SF metabolites similar regardless of GR dose, & much higher when taking SF; diurnal cycling obser.	[61]
2007	Cornblatt	Reduction mammaplasty	**x**				**x**				SF-rich beverage (containing 200 μmole SF)	8	200 μmol SF once	2.86 (SF)	Measured serum and mammary tissue levels of SF metabolites	[55]
2007	Gasper	Healthy	**x**					**x**			“standard” and “HG” broccoli florets microwaved gently to make soup	16	683 and 2296 μM SF measured	1.42/4.92 (SF)	Consumption of high glucosinolate broccoli resulted in up-regulation of several xenobiotic metabolizing genes in gastric mucosal tissue	[100]
2007	Myzak	Healthy			**x**			**x**			68 g broccoli sprouts (approximately 105 mg SF)	3	847 μmol SF if all converted from GR using author′s estimate; 136 μmol SF using our estimate	12.1 (auth)/1.9 (us) (SF)	HDAC activity was significantly inhibited in PBMC	[125]
2007	Rungapamestry	Healthy	**x**	**x**	**x**		**x**	**x**			150 g lightly cooked broccoli or fully cooked broccoli or a broccoli seed extract with added mustard seed	12	62 & 71.7 μmol GR/d or 2.7 μmol SF	0.89/1.0 (GR)	Estimated yield of SF was ~ 3-fold higher after consumption of lightly cooked broccoli than fully cooked broccoli. Meal matrix did not significantly influence the hydrolysis of GR and its excretion as SF	[126]
2008	Traka	Diagnosed with high-grade prostatic intraepithelial neoplasia (PIN)		**x**				**x**			150 g broccoli /d	22	79.3 μmol GR/d	1.13 (GR)	Showed complex change in signaling pathways associated with inflammation and carcinogenesis: Modified by GSTM1 genotype in broccoli feeding group	[79]
2008	Vermeulen	Healthy	**x**	**x**				**x**			200 g crushed raw or cooked broccoli	8	9.92 μmol SF or 61.4 μmol GR/d	0.14 (SF)/0.88 (GR)	10x greater bioavailability of SF w/ crushed, raw vs. microwaved broccoli	[127]
2009	Hanlon	Healthy	**x**					**x**			300 mL of homogenized raw broccoli w/ 3.9 mg SF, for 10 consecutive days	6	22 μmol SF/d	0.31 (SF)	Repeated intake of broccoli had no impact on the pharmacokinetic behavior or plasma levels of SF	[128]
2009	Riedl	Healthy	**x**					**x**			150, 175, and 200 g broccoli sprout homogenate mixed with daikon sprouts homogenate, once daily for 3 days	65	75 μmol/87.5 μmol /100 μmol SF/day (est)	1.07/1.25/1.43 (SF)	Increased Phase II enzyme expression in nasal lavage cells occurred in a dose-dependent manner	[76]
2009	Riso	Healthy (10 smokers and 10 nonsmokers)		**x**				**x**			200 g blanched broccoli x 10 d	20	200 μmol total ITC equivalents (presumed by us to be </=50 μmol GR	0.71 (GR)	↓ Strand breaks with broccoli diet in smokers/nonsmokers. ↓ oxidized purines only in smokers. Broccoli intake did not modify HDAC activity or IGF-I serum levels	[99]
2009	Yanaka	*H. pylori*–infected		**x**	**x**			**x**			GR-rich broccoli sprouts for 8 weeks	47	420 μmol GR (+ mryrosinase)	6 (GR)	Decreased levels of urease measured by the urea breath test and *H. pylori* stool antigen; decreased serum biomarkers of gastric inflammation	[116]
2010	Christiansen	Hypertensive, without diabetes & with normal cholesterol	**x**					**x**		**x**	10 g dried broccoli sprouts/d	40	259 μmol GR (measured)	3.7 (GR)	Daily ingestion of 10 g dried broccoli sprouts does not improve endothelial function in the presence of hypertension	[108]
2011	Bahadoran	Type 2 disbetes	**x**				**x**		**x**		5 g or 10 g broccoli sprouts powder (BroccoPhane) said to contain SF, daily for 4 weeks (we have assayed previously and found this not to be the case)	81	113 or 225 μmol SF/d based only on manufacturer′s claims	1.61/3.22 (SF)	Significant decrease in malondialdehyde, oxidized low density lipoprotein cholesterol, oxidative stress index & significant increase in total serum antioxidant capacity	[111]
2011	Clarke	Healthy non-smokers	**x**	**x**	**x**	**x**		**x**	**x**		68 g fresh broccoli sprouts or supplement purported to contain GR + Myr from 3 g dried broccoli sprouts	24	~ <220 μmol GR	3.1 (GR)	Consumption of fresh sprouts with active myrosinase provides 5x more bioavailable SF; SF and erucin metabolites are readily interconverted	[129]
2011	Egner	Healthy	**x**	**x**			**x**			**x**	SF-rich beverage (containing 150 μmole SF) or GR-rich beverage (containing 800 μmole GR) daily for 7 days.	69	800 μmol GR/d, 150 μmol SF /d	11.4 (GR)/2.14 (SF)	Bioavailability of SF (measured as urinary metabolites) was substantially greater with the SF-rich than GR rich beverage	[63]
2011	Healy	Healthy		**x**			**x**			**x**	561 mg BSE powder (200 μmol GR)	4	200 μmol GR/d	2.86 (GR)	Consumption of BSE showed inactivation of urinary MIF tautomerase activity in urine	[113]
2011	Hauder	Healthy male non-smoking (50-82 y.o.)		**x**				**x**			200 g of blanched, regular or selenium-fertilized broccoli, daily, for 4 weeks	76	broccoli contained either 133 or 226 μg/g of GR	0.87 or 1.48 (GR)	Dietary intake of selenium-fertilized broccoli increased serum selenium concentration, but affected neither glucosinolate concentrations in broccoli nor their metabolite levels in plasma and urine compared to regular broccoli.	[130]
2012	Bahadoran	Type 2 diabetes	**x**				**x**		**x**		10 g or 5 g broccoli sprouts powder (BroccoPhane) said to contain SF, daily for 4 weeks.	81	113 or 225 μmol SF/day based only on manufacturer′s claims	1.61/3.22 (SF)	Consumption of 10 g/d resulted in a significant decrease in serum insulin concentration and HOMA-IR	[112]
2012	Cramer	Healthy		**x**	**x**			**x**	**x**		2 g GR powder or 42 g fresh broccoli sprouts/d; once a week for 4 weeks	4	120 μmol GR/d or 70 μmol GR (fresh sprouts with active myrosinase)/d	1.71/1 (GR)	Adding fresh broccoli sprouts (as a source of myrosinase) to GR synergistically enhanced absorption and excretion	[131]
2012	Fahey	Healthy		**x**			**x**			**x**	200 μmol GR	131	200 μmol GR	2.86 (GR)	Extreme inter-individual range of conversion efficiencies (1–40% of administered dose of GR) attributed to differences in microbiomes as well as circadian rhythm; within-individual differences were less pronounced	[65]
2012	Kensler	Healthy	**x**	**x**			**x**			**x**	Cross-over design: GR-rich beverage (800 μmol GR) → SF-rich beverage (150 μmol SF)/SF-rich beverage (150 μmol SF) →GR-rich beverage (800 μmol GR)	50	800 μmol GR/day, 150 μmol SF /d	11.4 (GR)/2.14 (SF)	Statistically significant increases in the levels of excretion of glutathione-derived conjugates of benzene and acrolein, but not crotonaldehyde in groups receiving SF-rich, GR-rich beverages or both compared to preintervention values	[77]
2012	Mirmiran	Type 2 diabetes	**x**		**x**		**x**		**x**		5 or 10 g/d of BroccoPhane powder (BSP), reported to be rich in SF, -- daily x 4 wks (we have assayed previously and found this not to be the case)	81	113 or 225 μmol SF/day based only on manufacturer′s claims	1.61/3.22 (SF)	Sera of BSP treatment groups showed ↓ hs-CRP, and non-significant ↓ IL-6 and TNF-α	[110]
2012	Saha	Healthy	**x**	**x**				**x**			Broccoli soups produced from fresh or frozen broccoli florets	18	4.16 mg SF/serv (23.5 μmol) or 18.6 mg GR/serv. (42.7 μmol)	0.33 (SF)/0.61 (GR)	SF bioavailability was~10x higher in fresh compared to frozen broccoli	[132]
2013	Armah	10-y CVD risk profile		**x**				**x**			400 g standard or “HG” broccoli or placebo vegetable (peas) for 12 wk	54	6.9 or 21.6 μmol GR/g dry wt broccoli	1.48/4.63 (GR)	No significant differences on markers of CVD risk	[82]
2013	Meyer	Healthy	**x**					**x**			SF-rich homogenate prepared from 200 g BroccoSprouts (assayed for SF) daily for 3 consecutive days	12	100 μmol SF/day (our est.)	1.43 (SF)	Homogenate significantly increased secretory leukocyte protease inhibitor levels in nasal lavage fluid	[94]
2013	Poulton	Healthy	**x**				**x**				BSE (450 µmol SF/d x 7 d delivered in cheese-based soup	23	450 μmol SF/d	6.43 (SF)	No effect on CYP3A4 activity	[44]
2014	Bahadoran	Type 2 diabetic with *H. pylori* infection	**x**				**x**		**x**		6 g/d of Cyvex broccoli sprouts powder (BSP), reported to be rich in SF, (we have assayed previously and found this not to be the case), daily, in combination with other drugs, for up to 28 d	86	Maximum possible is 135 μmol SF/day	1.93 (SF)	BSP ↓ *H. pylori* load in diabetic patients, but no effect on gastric inflammatory markers	[117]
2014	Baier	Healthy			**x**			**x**			34, 68 or 102 g Broccoli sprouts	8	170, 340, and 680 μmol GR/day	2.4/4.8/9.6 (GR)	Dose dependent elevation in LTR (long terminal repeats) mRNA & histone acetylation in circulating leukocytes	[133]
2014	Egner	Healthy	**x**	**x**			**x**			**x**	broccoli sprouts beverages containing GR-rich and SF-rich powders (600 μmol GR + 40 μmol SF), daily, for 150 days	267	600 umol GR/day + 40 μmol SF/day	8.57 (GR) + 0.57 (SF)	Significant increases in the levels of excretion of the glutathione-derived conjugates of benzene and acrolein in urine in broccoli sprout beverage treatment group	[78]
2014	Heber	Healthy	**x**				**x**			**x**	1.25 g BSE suspended in juice (100 μmol SF/d x 4 d)	29	100 μmol SF/d	1.43 (SF)	Subjects challenged w/ repeated nasal diesel exhaust particles: WBC decreased 54% by daily BSE	[91]
2014	Noah	Healthy	**x**					**x**			smokers/nonsmokers - broccoli sprout homogenate	16/35	100 μmol SF/d (est)	1.43 (SF)	Post BSH, live attenuated influenza virus-induced inflammatory markers reduced; NQO1 increased, in smokers but not non-smokers	[92]
2014	Singh	♂,13–27 y.o. with moderate to severe Autism Spectrum Disorder	**x**			**x**				**x**	50 - 150 µmol SF/d x 18 weeks; dosed by BW	44	50 μmol SF/d - 150 μmol SF/d	0.71 - 2.14 (SF)	SF showed substantial declines (improvement of behavior) in Aberrant Behavior Checklist and Social Responsiveness Scale scores	[87]
2015	Alumkal	Prostate cancer	**x**			**x**				**x**	SF-rich broccoli sprout extract; 200 μmol/d x 20 weeks	20	200 μmol SF/d	2.86 (SF)	Did not lead to ≥50% PSA declines	[103]
2015	Armah	10 Year cardiovascular risk profile of between 10 and 20%		**x**				**x**			400 g standard or “HG” broccoli or placebo vegetable (peas) for 12 wk	130	6.9 or 21.6 μmol GR/g dry wt broccoli	1.48/4.63 (GR)	High glucoraphanin (HG) broccoli diet ↓ plasma LDL-C more than standard broccoli	[85]
2015	Atwell	Abnormal mammograms; scheduled for breast biopsy			**x**		**x**		**x**		broccoli seed extract containing GR (BroccoMax); [we have assayed previously and found the myrosinase to be inactive and the GR titer to not be as represented], 2 capsules, 3x/d, x 2-8 wk	54	180 mg GR/day (= 413 μmol GR/d)	5.9 (GR)	↓ PBMC HDAC activity; pre-to-post changes in Ki-67 and HDAC3; NSD in tissue biomarkers between placebo and treatment group	[98]
2015	Atwell	Healthy	**x**	**x**		**x**		**x**		**x**	fresh broccoli sprouts (containing 200 μmol GR) or SF-rich BSE (containing 200 μmol SF) daily, consumed every 12 h.	20	200 μmol SF/d; 200 μmol GR/d	3.33 (SF); 3.33 (GR)	3 x higher SF metabolite levels in plasma and urine in sprout consumers compared to SF-rich BSE consumers	[57]
2015	Brown	Moderate asthma	**x**			**x**				**x**	440 mg SF-rich BSE (100 μmol SF/d x 14 d)	45	100 μmol SF/d	1.43 (SF)	Individuals in whom SF treatment enhanced the forced expiratory volume response to methacholine, had increased expression of Nrf2-regulated antioxidant and anti-inflammatory genes in peripheral blood mononuclear cells: SF treatment resulted in significant reduction in airway resistance and increased small airway luminal area	[73]
2015	Chang	*H. pylori*	**x**			**x**			**x**		SF capsule twice daily for 4 weeks.	67	2 mg SF/day (11.3 μmol SF/d) [we do not trust this company′s representation of dose]	0.16 (SF)	No significant difference in urea breath test values or ammonia concentration; SF did ↓ gastric mucosal malondialdehyde but not glutathione levels	[134]
2015	Cipolla	Prostate cancer patients post- radical prostatectomy	**x**			**x**			**x**		SF tablets (2 tablets containing 10 mg stabilized SF extracted from broccoli seeds, 3 times a day) for 6 months	78	60 mg SF /day (339 μmol SF/d)	4.83 (SF)	Decreased prostate-specific antigen (PSA) levels	[102]
2015	Fahey	Healthy		**x**	**x**	**x**	**x**		**x**	**x**	50, 69, 100, 200, 230 μmol GR in various dose forms including comparing commercial tablets to investigator-prepared materials and GR with active myrosinase	20	50, 69, 100, 200 & 230 μmol GR	0.7/1.0/1.4/2.9/3.3 (GR)	Inter- and intra-individual variabilities, when GR delivered in teas, juices, or gelatin capsules; established effect of adding active myrosinase to the “dose”	[68]
2015	Kikuchi	Diagnosis of fatty liver with elevated liver function markers		**x**							3 broccoli sprout capsules (containing 30 mg GR) for 2 months	55	69 μmol GR/d	1 (GR)	↓ serum ALT, AST, γ-GTP	[118]
2015	Medina	Healthy			**x**			**x**			30 g or 60 g of broccoli sprouts	24	~117 or 234 μmol GR (measured)/d as well as much smaller amounts of SF (measured)	1.67/3.34 (GR)	Broccoli sprouts modulated excretion of biomarkers linked to inflammation and vascular reactions without exerting a significant influence on the oxidation of phospholipids	[135]
2015	Shiina	Schizophrenia	**x**			**x**			**x**		30 mg GR/d x 8 weeks	10	30 mg GR/d (69 μmol GR/d)	1 (GR)	Mean score in the Accuracy component of the One Card Learning Task increased significantly after the trial.	[136]
2015	Ushida	Healthy		**x**		**x**			**x**		dried broccoli sprout capsules (3 or 6 capsules rich in GR)	21	68.7 or 137.4 μmol GR/day	0.98/1.96 (GR)	Serum activities of GST and NQO1 were dose-dependently and synchronously elevated	[114]
2016	Bauman	Healthy		**x**						**x**	150 μmol SF oral swallowed or held in mouth, or 600 μmol GR swallowed	10		8.57 (GR)/2.14 (SF)	Clinical support for good mucosal bioactivity and pharmacodynamic activity	[72]
2016	Doss	Sickle cell disease (SCD)	**x**	**x**				**x**			broccoli sprout homogenate (BSH) made from 50, 100, or 150 g fresh BroccoSprouts	16	250, 500, or 750 μmol GR [calculated maximum delivery]	3.6/7.1/10.7 (GR)	Homogenate is safe in SCD subjects; only modest changes in NRF2-mediated gene expression	[74]
2016	Duran	Healthy			**x**			**x**			Homogenate prepared from 111 g BroccoSprouts once daily for 3 days	16	555 μmol GR/day	7.9 (GR)	On last treatment day, subjects were exposed to ozone with intermittent moderate exercise to induce airway inflammation: Homogenate did not induce expression of antioxidant genes in blood and nasal epithelial cells	[97]
2016	Müller	Healthy		**x**				**x**			Broccoli sprout homogenate shake prepared from 200 g sprout (about 100μmol of SF per dose) or alfalfa sprout homogenate for control, for 4 days	42	100 μmol SF/day (est.)	1.43 (SF)	BSH supplementation increased live attenuated influenza virus-induced granzyme B production in NK cells compared to control	[96]
2016	Sudini	Asthma		**x**				**x**			100 g fresh broccoli sprouts (BS) /d or 100 g of alfalfa sprouts (placebo) x 3 consecutive d	40	no GR or SF measurements made or imputed; max poss. expected to be 500 μmol GR/d	7.14 (GR)	No induction of cytoprotective antioxidant genes in either PBMCs or nasal epithelial cells or ↓ oxidative stress and inflammatory markers in urine and serum; no improved lung function.	[95]
2016	Wise	COPD	**x**			**x**				**x**	SF extracted from broccoli sprouts (25 μmol/150 μmol) daily for four weeks	89	25 μmol SF/d; 150 μmol SF/d	0.36 & 2.14 (SF)	SF did not stimulate expression of Nrf2 target genes or have an effect on levels of other anti-oxidants or markers of inflammation.	[105]
2017	Axelsson	Diabetics (well regulated, and dysregulated)		**x**			**x**		**x**		5410 ppm of SF x 5 g/d	97	153 μmol SF/d	2.18 (SF)	SF reduced fasting blood glucose (hepatic gluconeogenesis) & glycated hemoglobin (HbA1c) in obese patients with dysregulated T2D	[86]
2017	Davidson	Osteoarthritis		**x**				**x**			100 g high glucosinolate (HG) broccoli/d x 14 d	40	180 μmol GR/d	2.57 (GR)	ITCs detected in synovial fluid of HG group, but not the low glucosinolate group.	[59]
2017	Fahey	Healthy	**x**			**x**	**x**		**x**	**x**	94.4 μmol SF/d or 200 μmol α-cyclodextrin enrobed SF/d	10	94.4 μmol SF/d	1.35 & 2.86 (SF)	PK and tolerance of α-cyclodextrin enrobed SF (for theoretical stabilization) was compared to that of SF along, in the commercial “stabilized SF” product Prostaphane™	[66]
2018	Sedlak	Healthy	**x**			**x**				**x**	100 μmol SF/d x 7 d taken in 2 gel caps per day	9	100 μmol SF/d	1.43 (SF)	Correlation between blood and thalamic GSH post- and pre-SF treatment ratios and a consistent increase in brain GSH levels (7 Tesla MRI)	[81]
2018	Tahata	Melanoma	**x**				**x**			**x**	Broccoli sprout extract-SF standardized for 50, 100, or 200 µmol SF for 28 days	17	50 μmol /100 umol/200 μmol SF/day	0.71/1.43/2.86 (SF)	Oral BSE-SF is well tolerated at 50, 100, and 200 μmol /day attaining blood plasma and skin biopsy levels reasonable for pharmacodynamic action	[104]
2018	Bent	Children with ASD and related neurodevelopmental disorders			**x**				**x**		6 to 15 Avmacol tablets (222 μmol GR to 555 μmol GR) daily for 12 weeks, depending on BW	15	222 umol GR/day - 555 umol GR/day	3.17 - 7.93 (GR)	Mean scores on both symptom measures showed improvements (decreases) over the study period, which was correlated with urinary metabolites	[120]
2018	Housley	Healthy			**x**			**x**			fresh broccoli sprouts (containing 200 μmol GR)	10	200 μmol GR/d	2.86 (GR)	Untargeted metabolomic screen of human plasma following consumption of fresh broccoli sprouts	[137]
2018	Okunade	Healthy		**x**	**x**			**x**			200 g raw, cooked broccoli ± 1 g mustard powder	12	62, 32, or 257 μmol GR/d based on conversion of author′s dry wt., to (our) fresh wt. basis	0.88/0.45/3.7 (GR)	↑ urinary SF-NAC when cooked broccoli consumed with mustard powder	[138]
2018	Sivapalan	Healthy		**x**				**x**			300 g Myb28B/B (standard) or Myb28B/V (Beneforte) or Myb28V/V broccoli soup; single dose	10	84, 280, or 452 μmol GR/d	1.2/4.0/6.5 (GR)	Three different Myb28 genotypes of broccoli related with delivery of sulforaphane to the systemic circulation.	[52]
2019	Chartoumpekis	Healthy women	**x**	**x**			**x**			**x**	Broccoli sprouts beverages containing GR-rich and SF-rich powders (600 μmol GR + 40 μmol SF), daily, for 150 days	45	600 umol GR/day + 40 μmol SF/day	8.57 (GR) + 0.57 (SF)	Measurement of thyroglobulin, TSK, free thyroxine, and others	[62]
2019	Lopez Chillon	Healthy, overweight			**x**			**x**			30 g/d of fresh broccoli sprouts, for 10 wks followed by 10 wks of washout	40	117 μmol GR/d (measured)	1.67 (GR)	Reduced IL-6 and CRP following broccoli sprout consumption	[109]
2019	Chen	Healthy	**x**	**x**			**x**			**x**	Broccoli sprout beverage (contained GR and SF), full dose or half dose or fifth dose, daily for 10 consecutive days	170	600 μmol GR + 40 µmol SF or 300 μmol GR + 20 µmol SF or 125μmol GR + 8 µmol SF/day	600/40, 300/20, or 125/8 (GR + SF)	Benzene mercapturic acids in urine was increased in high dose treated group, but not in half dose and one-fifth dose	[69]
2019	Fahey	Healthy			**x**	**x**			**x**		Avmacol - (GR with active myrosinase; 6 tablets211 μ, mol GR, single dose, n=20); and 8 Avmacol tablets (369 μmol GR), on 4 separate days, enteric-coated and not-coated	20 & 16	211 and 369 μmol GR/day	3.01/5.27 (GR)	Gastric acidity somewhat attenuates activity of oral myrosinase reducing conversion of GR to SF, and thus SF bioavailability; cytoprotection, antioxidant and detoxification gene expression increased with increasing SF bioavailability.	[67]
2019	Traka	Men with low to intermed. risk prostate cancer		**x**				**x**			broccoli	61	72 - 492 μmol GR	1 - 7 (GR)	Affected gene expression in the prostates of men under active surveillance, consistent with prevention	[80]
2020	Bauman	Head and neck cancer survivors			**x**				**x**		Avmacol 50 or 100 mg GR (115 or 230 μmol GR)	36	115 or 230 μmol GR	1.6-3.3	Ongoing; “Preventing Recurrence in Patients With Tobacco-Related Head and Neck Squamous Cell Cancer”	NCT03182959
2020	Kim	*H. pylori* infected adults (18-75 y.o.)	**x**								broccoli sprout extract	360		--	Ongoing; “The Effect of Broccoli Sprout Extract and Probiotics for Eradication of Helicobacter Pylori”	NCT03220542
2020	Bauman	Smokers			**x**				**x**		Avmacol - 4 or 8 tablets/d	61	(our calculation) ~ 138 and 275 μmol GR	2.0/4.0 (GR)	Ongoing; Decreasing Toxicity in Heavy Smokers	NCT03402230
2020	Dickerson	Adults (18-65 y.o.) with schizophrenia			**x**	**x**			**x**		Avmacol	64	weight-based, about 1.4 μmol/kg BW	1.42 (GR)	Ongoing; amelioration of symptoms of schizophrenia	NCT02810964
2020	Hua/Davis	Children (3-15 y.o.) on the autism spectrum			**x**	**x**			**x**		Avmacol	110		--	Ongoing; amelioration of symptoms of autism spectrum disorder (ASD)	NCT02879110
2020	Hua/Davis	Adults with 1st episode or early onset schizophrenia			**x**	**x**			**x**		Avmacol	180		--	Ongoing; amelioration of symptoms of schizophrenia	NCT02880462
2020	Johnson	Young adults (13-30 y.o.), on the autism spectrum			**x**	**x**			**x**		Avmacol	45	weight-based, about 1.5 μmol/kg BW	1.47 (GR)	Ongoing; amelioration of symptoms of autism spectrum disorder (ASD)	NCT02677051
2020	Li	Veterans with allergic rhinitis	**x**	**x**							broccoli sprout extract	475	Phase 2 RCT; BSE paired with fluticasone or normal saline spray	--	Ongoing; “Effects of Broccoli Sprout Extract on Allergy Rhinitis”	NCT 0288 5025
2020	Politte	Young men (13-30 y.o.), on the autism spectrum			**x**	**x**			**x**		Avmacol	48	weight-based, about 1.4 μmol/kg BW	1.4 (GR)	Ongoing; amelioration of symptoms of autism spectrum disorder (ASD)	NCT 0290 9959
2020	Tex Tech	Doxirubicin-naïve women with breast cancer			**x**				**x**		Avmacol (2 - 8 tablets/d) x 12 weeks; weight based	70	68.8 - 275 μmol GR	1 - 3.9 (GR)	Ongoing; “Effects of the SF on doxorubicin-associated cardiac dysfunction”	NCT 0393 4905
2020	Wang	Adults at risk for psychosis			**x**	**x**			**x**		Chinese commercial “GR + Myros. “ supplement (Zhiyinguosu)	300	52 wk, daily about 411 μmol GR with active myrosinase	5.9 (GR)	Ongoing; 1° planned outcome -- conversion to psychosis	NCT 0393 2136
2020	Wu	1st episode or early onset schizophrenia (SZ)			**x**				**x**		Avmacol	180		--	Ongoing; “A 6-month Study to Evaluate SF add-on Effects in Treatment of SZ”	NCT 0288 0462
2020	Yuan	Former Smokers			**x**				**x**		Avmacol	72	120 μmol 2x/d	3.4 (GR)	Ongoing; lung cancer prevention in former smokers	NCT 0323 2138
2020	Zandberg	Head & neck cancer patients post-curative treatment			**x**	**x**			**x**		Avmacol; escalating daily doses, from 2 to 4 to 8 tabs per day for a month each	36	69, 138, or 275 μmol GR	1/2/3.9 (GR)	Ongoing	NCT 0326 8993
2020	Zimmerman	Children (3-12 y.o.), on the autism spectrum			**x**	**x**			**x**		Avmacol	60	weight-based, about 2.2 μmol/kg BW	2.2 (GR)	Ongoing; amelioration of symptoms of autism spectrum disorder	NCT 0256 1481

^1^ Year published, if published results exist. In cases where it appears that the trial is well underway and/or when we have queried the P.I., we have included “pending” trials that are listed on clincaltrials.gov. In such cases “year pub′d” is arbitrarily given as 2020. ^2^ Compound(s) delivered are either sulforaphane (SF), glucoraphanin (GR) or GR with added, active, myrosinase enzyme (GR + Myr) ^3^ Delivery formats are: Tablets or capsules (gelatin or vegan/vegetable gel-caps) - “Tabs/caps”; powders that may be added to juices, water, or other food products - “Powder/Other”; fresh broccoli or broccoli sprouts, either cooked in various ways as indicated, or raw -- “Fresh” ^4^ When products are commercially supplied, there is reason for concern over use solely of manufacturers′ reported compound (GR or SF) titer. Many investigators appropriately measure compound concentration themselves and where we have included studies that do not do so, we have attempted to so note. ^5^ As above, we have attempted to note cases where titer is suspect and in some cases we have tested the products used (purchased commercially ourselves, so likely other “lots”), and found them to be vastly different in content or quality, from indications made in the sales literature for those products. Other abbreviations used herein -- BSE, broccoli sprout extract; BSP, broccoli sprout powder; GS, glucosinolate; ITC, isothiocyanate; ASD, autism spectrum disorder; SZ schizophrenia; RCT, randomized control trial ^6^ Assumptions made in calculations are that if fed homogenized fresh broccoli or broccoli sprouts, they were getting SF and it was in many cases impossible to even guess how much, if any. Thus, if given fresh broccoli or broccoli sprouts and titer not provided in reference, our assumption is that sprout titer was ≤5 μmol GR/g sprouts, and subjects were ingesting “GR + Myr”. ^7^ In cases where the authors did not indicate dosage in μmol/kg body weight (BW), we have made those calculations using the *a priori* assumption of a 70 kg BW.

## Supplementary Materials

The following are available online. Table S1: Summary of literature reporting oral dosing of mice with sulforaphane, Table S2: Summary of literature reporting intraperitoneal dosing of mice with sulforaphane, Table S3: Summary of literature reporting topical administration of sulforaphane to mice, Table S4: Summary of literature reporting oral dosing of rats with sulforaphane.

## Dedication

The lives and careers of the three senior authors on this review have been affected profoundly by the friendship and mentoring provided by the late Paul Talalay MD (1923–2019). His visionary precept that rigorous, quantitative sciences would serve as the foundation for the discovery, isolation, evaluation, and clinical translation of natural products for disease prevention, especially those isolated from vegetables, has guided the scientific inquiries discussed in this article. He literally planted the seeds for the continuing evolution of broccoli, and specifically sulforaphane, as an effective agent for reducing the burdens of disease in humans as reviewed in his “Reflections” writing in the *Journal of Biological Chemistry* [146] and as reflected in a literature that now exceeds 3000 papers. His scientific legacy continues to expand and will be nurtured by many.
